# Combination of WEE1 Inhibitor and Vitamin K2 Enhances Therapeutic Efficacy in Chronic Myeloid Leukemia

**DOI:** 10.1002/cai2.70024

**Published:** 2025-08-28

**Authors:** Seiichi Okabe, Yuya Arai, Akihiko Gotoh, Daigo Akahane

**Affiliations:** ^1^ Department of Hematology Tokyo Medical University Tokyo Japan

**Keywords:** chronic myeloid leukemia, vitamin K2, WEE1 inhibitor

## Abstract

**Background:**

Chronic myeloid leukemia (CML) is a clonal malignancy propelled by the *BCR::ABL1* fusion gene originating from the Philadelphia chromosome. This gene activates ABL tyrosine kinase, which enhances the survival of leukemic cells. Although tyrosine kinase inhibitors (TKIs) have significantly advanced the treatment of CML, resistance to these inhibitors presents a substantial hurdle. Consequently, novel therapeutic strategies targeting resistance mechanisms independent of *BCR::ABL1* are urgently needed.

**Methods:**

This study investigated the potential impact of combining WEE1 inhibitors, particularly MK‐1775, with vitamin K2 (VK2) in treating CML. To analyze differentially expressed and spliced transcripts in CML, we examined mRNA profiles from peripheral blood mononuclear cells of five patients with CML (during chronic and blast phases) and five healthy controls. The samples were analyzed using deep sequencing. Differential expression analyses were performed using RaNA‐Seq and Heatmapper, the latter of which was designed for complex data set visualizations.

**Results:**

WEE1 controls the G2/M checkpoint to prevent early mitosis, and blocking it increases the cytotoxicity of agents that damage deoxyribonucleic acid, especially in cancers lacking p53. VK2, a micronutrient, exerts anticancer effects against various malignancies. Gene expression studies have indicated that PKMYT1 expression is elevated in CML but not WEE1 cells. MK‐1775 successfully halted the growth of both standard and TKI‐resistant CML cell lines by triggering apoptosis via caspase 3/7 activation. VK2 reduced the viability of CML cells and increased cytotoxicity. A combined regimen of MK‐1775 and VK2 markedly decreased colony growth, disrupted mitochondrial membrane potential, and increased death in CML cells, including those resistant to TKIs.

**Conclusions:**

The results suggest that a combination of MK‐1775 and VK2 represents a potentially effective treatment strategy for CML, especially in drug‐resistant cases.

## Graphical abstract

Combining the WEE1 inhibitor MK‐1775 with vitamin K2 enhanced cytotoxicity and apoptosis in Chronic myeloid leukemia(CML) cells, including TKI‐resistant strains. This combination therapy shows promise as a novel approach to overcome drug resistance in CML.

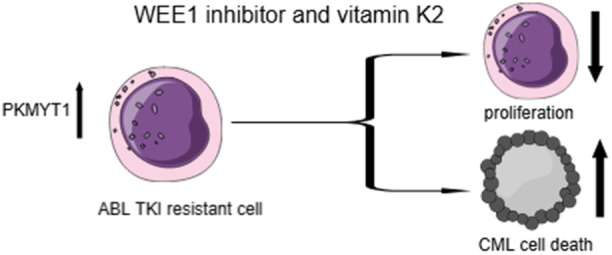

**Abbreviations**
ANOVA: analysis of variance
AP: accelerated phase
BP: blastic phase
CI: combination index
CML: chronic myeloid leukemia
CP: chronic phase
FCS: fetal calf serum
GEO: Gene Expression Omnibus
LDH: lactate dehydrogenase
MMP: mitochondrial membrane potential
mRNA: messenger RNA
Ph: Philadelphia chromosome
PKMYT1: membrane‐associated tyrosine‐ and threonine‐specific cdc2‐inhibitory kinase
qRT‐PCR: quantitative real‐time reverse transcription‐polymerase chain reaction analysis
TKI: tyrosine kinase inhibitor
VK1: vitamin K1
VK2: vitamin K2
WEE1: WEE1 G2 checkpoint kinase
WEE2: WEE1‐like protein kinase 2

## 1 Background

Chronic myeloid leukemia (CML) is a form of cancer characterized by abnormal growth and proliferation of white blood cells, primarily affecting the bone marrow and bloodstream [1]. CML is caused by balanced reciprocal translocation between Chromosomes 9 and 22, designated t(9;22)(q34;q11). This translocation results in the formation of the Philadelphia chromosome (Ph) [2]. This fusion gene causes persistent activation of ABL tyrosine kinase, which plays a crucial role in the survival and growth of leukemic cells [3]. The clinical progression of CML is categorized into three separate phases: chronic phase (CP), accelerated phase (AP), and blastic phase (BP). These phases represent the natural progression of the disease when no effective treatment is available [4]. Tyrosine kinase inhibitors (TKIs) have significantly advanced the treatment of CML, resulting in a 5‐year survival rate > 80% [5].

The standard first‐line treatment for CML includes ABL TKIs, such as imatinib, dasatinib, nilotinib, and bosutinib [6]. Although TKIs have revolutionized CML treatment, resistance and intolerance remain major challenges, necessitating alternative approaches. Some patients develop resistance to TKI therapy even after successful inhibition of the *BCR::ABL1* fusion gene [7]. For patients with CML, novel therapeutic approaches that address *BCR::ABL1*‐independent resistance mechanisms are essential. The ongoing research aims to identify and target the pathways involved in TKI resistance, paving the way for improved treatment options.

WEE1 plays a key role in cell cycle control, particularly at the G2/M checkpoint, by inhibiting cyclin‐dependent kinase 1 and preventing early entry into mitosis [8]. The WEE1 kinase family comprises three serine/threonine kinases, each encoded by separate genes: *WEE1* (WEE1 G2 checkpoint kinase), *PKMYT1* (membrane‐associated tyrosine‐ and threonine‐specific cdc2‐inhibitory kinase), and *WEE2* (WEE1‐like protein kinase 2) [8]. WEE1 and PKMYT1, key regulators of mitotic entry, are essential in DNA damage response pathways. These proteins ensure proper cell cycle progression and enable DNA repair before cells proceed into mitosis [9, 10]. WEE1 kinase inhibitors have garnered considerable attention in cancer therapy because of their ability to exploit weakened DNA damage response pathways in cancer cells [10]. Recent preclinical and clinical studies on human carcinoma have investigated the use of WEE1 inhibitors such as MK‐1775 to enhance the cytotoxic effects of DNA‐damaging agents [11]. This process results in mitotic catastrophe and cell death, particularly in cells with deficient p53 functionality, a common genetic alteration in various cancer types [8].

The vitamin K family comprises two important naturally occurring micronutrients: vitamin K1 (VK1) and vitamin K2 (VK2). These vital nutrients are sourced from various foods: VK1 is mainly found in green leafy vegetables and algae, whereas VK2 is abundant in animal‐based products and fermented foods [12]. VK2, an essential nutrient, is important for various physiological functions and may possess cancer‐fighting properties by affecting several signaling pathways involved in cancer progression [13].

In the present study, we aimed to investigate the potential synergistic effects of combining WEE1 inhibitors and VK2 for CML treatment. By examining the molecular mechanisms involved and evaluating the effect of the combination on cancer cell survival, we sought to determine its potential as a novel therapeutic approach to overcome drug resistance and improve treatment outcomes in CML.

## 2 Methods

### 2.1 Reagents

This study used only analytical‐grade reagents. MK‐1775, a WEE1 inhibitor, was obtained from Selleck Chemicals (Houston, TX, USA). Stock solutions of MK‐1775 were prepared in dimethyl sulfoxide and stored at −20°C. VK2 (menatetrenone) was provided by Eisai Co. Ltd. (Bunkyo‐ku, Tokyo, Japan) and diluted in culture medium. All additional reagents were supplied by Merck KGaA (Darmstadt, Germany).

### 2.2 Cell Culture

The CML cell line K562 was obtained from the American Type Culture Collection (ATCC, Manassas, Virginia, USA). Several other cell lines have been established, including Ba/F3 cells with the T315I mutation, Ba/F3 cells transfected with wild‐type *BCR::ABL1*, Ba/F3 cells resistant to ponatinib (Ba/F3 PR), K562 cells resistant to ponatinib (K562 PR), and the Ph‐positive cell line SK‐9 carrying the T315I mutation [14, 15, 16]. The cell lines were cultured in Roswell Park Memorial Institute 1640 medium supplemented with 10% fetal calf serum (FCS), 1% penicillin–streptomycin, and 1% glutamine. Cells were incubated at 37°C in a humidified atmosphere containing 5% CO_2_.

### 2.3 Data Collection and Processing

To elucidate the differentially expressed and spliced transcripts in CML, we investigated the messenger ribonucleic acid (mRNA) profiles of peripheral blood mononuclear cells. We used samples from five patients with CML in both the CP and BP groups as well as five healthy individuals. These samples were previously analyzed by deep sequencing using the Illumina NextSeq. 500 platform (data set GSE100026). The data set, labeled GSE100026, is available through the Gene Expression Omnibus (GEO) database and can be accessed at https://www.ncbi.nlm.nih.gov/geo/query [17]. The GSE4170 data set provides valuable insights into the molecular landscape of CML, a hematopoietic stem cell malignancy characterized by distinct biological and clinical phases. In the GSE4170 study, gene expression profiling using DNA microarrays was performed on 91 CML patient samples, including 42 in CP, 17 in AP, and 32 in BP [18]. Differential expression analysis of RNA‐Seq count data was performed using EdgeR, an R software package, according to the instructions outlined in the user manual. RNA‐Seq data were visualized using the open bioinformatics web tool RaNA‐Seq (accessible at http://ranaseq.eu), and Heatmapper, an advanced bioinformatics application, was used to generate a heatmap [19, 20]. The proposed tool is specifically designed to visualize intricate data sets in scientific research.

### 2.4 Cell Proliferation Assay

Cells were seeded in 96‐well plates at concentrations of 4 × 10^3^ or 5 × 10^3^ cells per well and treated with MK‐1775 and VK2 for 72 h. Cell viability was assessed using three methods: CellTiter‐Glo Luminescent Cell Viability Assay Kit (Promega Corporation, Madison, WI, USA), Cell Counting Kit‐8 (Dojindo Laboratories, Mashikimachi, Kumamoto, Japan), and trypan blue staining assay (Bio‐Rad, Hercules, CA, USA). The assays were performed according to the manufacturers' protocols. After 72 h of incubation, the samples were analyzed using either an EnSpire Multimode Plate Reader (PerkinElmer, Waltham, MA, USA) or TC10 Automated Cell Counter (Bio‐Rad). The proliferation rate was expressed as a percentage relative to that of the untreated control cells. Quantitative determination of synergism or antagonism using the combination index (CI) was performed using the Chou–Talalay method [21].

### 2.5 Determination of Caspase 3/7 Activities

The Caspase‐Glo 3/7 Assay Kit (Promega) was used to assess caspase activity in CML cells following the manufacturer's instructions. CML cells were treated with the specified doses of MK‐1775 and VK2. Following a 48‐h incubation period, an equivalent volume of Caspase‐Glo 3/7 reagent was added to each well. An EnSpire Multimode Plate Reader (PerkinElmer) was used to measure luminescence and thus quantify caspase 3/7 activity.

### 2.6 Cytotoxicity Assays

CML cells were treated with specified concentrations of MK‐1775 and VK2. To assess cytotoxicity, lactate dehydrogenase (LDH) release from damaged cells was measured using a Cytotoxicity LDH Assay Kit, which employs water‐soluble tetrazolium salt produced by Dojindo Laboratories. After 48 h of treatment, LDH levels were determined using an EnSpire Multimode Plate Reader (PerkinElmer) according to the manufacturer's guidelines.

### 2.7 Colony Formation Assay

Colony formation was evaluated using MethoCult Express (Catalog #04437; STEMCELL Technologies, Vancouver, BC, Canada), a methylcellulose‐based culture medium, according to the manufacturer's protocol. Briefly, 1 × 10^2^ K562 or K562 PR cells were seeded in MethoCult Express medium containing defined concentrations of MK‐1775 and/or VK2. The plates were incubated at 37°C in a 5% CO_2_ environment for 7 days. The colonies were counted, and images were taken using an EVOS FL Digital Inverted Fluorescence Microscope (Thermo Fisher Scientific Inc., Waltham, MA, USA). The experiments were conducted in triplicate, and the results are expressed as mean values with standard errors.

### 2.8 Mitochondrial Membrane Potential Assay

The mitochondrial membrane potential (MMP) was assessed using a mitochondrial staining kit (Merck KGaA) according to the manufacturer's protocol. The cells were then treated with 500 nM MK‐1775 and 10 µM VK2 for 48 h. After incubation, JC‐1 monomers and aggregates were analyzed using an EnSpire Multimode Plate Reader (PerkinElmer).

### 2.9 Short‐Hairpin RNA Transfection

A lentiviral vector encoding short‐hairpin RNA (shRNA) targeting *PKMYT1* was used, along with a control shRNA vector, both obtained from VectorBuilder Japan Inc. (Yokohama, Kagawa, Japan). K562 cells were seeded in six‐well plates and cultured in RPMI 1640 medium supplemented with 8 µg/mL polybrene (hexadimethrine bromide; Merck KGaA) for 24 h. Lentiviral transduction was performed in accordance with the manufacturer's instructions. After 24 h of incubation, the culture medium was replaced, and *PKMYT1* expression was subsequently evaluated by quantitative reverse transcription PCR (RT‐qPCR).

### 2.10 RT‐qPCR Analysis

The total RNA was extracted from K562 cells using an RNAqueous‐4PCR Kit (Life Technologies Japan KK, Minato‐ku, Tokyo, Japan) according to the manufacturer's instructions. First‐strand cDNA was synthesized using a First‐Strand cDNA Synthesis Kit (OriGene Technologies, Rockville, MD, USA). Quantitative real‐time RT‐PCR was conducted using the LightCycler 2.0 system (Roche Diagnostics GmbH, Minato‐ku, Tokyo, Japan). Gene‐specific primers were obtained from Takara Bio Inc. (Otsu, Shiga, Japan). Gene expression was quantified using the SYBR Green PCR Kit (Roche) in accordance with the manufacturer's protocol.

### 2.11 Cell Cycle Analyses

The cell cycle distribution was assessed using the BD Cycletest Plus DNA Reagent Kit (BD Biosciences, Mountain View, CA, USA), following the manufacturer's protocol. K562 cells transfected with shRNA were cultured in standard medium for 24 h before analysis. The DNA content was then evaluated using a BD FACSVerse flow cytometer (BD Biosciences) and quantified using BD FACSuite software (BD Biosciences).

### 2.12 Statistical Analyses

GraphPad Prism 10 software (GraphPad Software, San Diego, CA, USA) was used for all data analysis. The results are expressed as the mean ± standard deviation, and each experiment was performed three times. A paired Student's *t*‐test was used to assess statistical significance between the two groups. When a single group was designated as the control, a one‐way analysis of variance (ANOVA) followed by the Dunnett post hoc test was applied, with an alpha level of 0.05 and *n* ≥ 3. Statistical significance is indicated by *p < 0.05, **p < 0.01, ***p < 0.001, and ****p < 0.0001.

## 3 Results

### 3.1 WEE1 Expression in CML Cells and MK‐1775 Inhibited Ph‐Positive Cells

**Figure 1:**
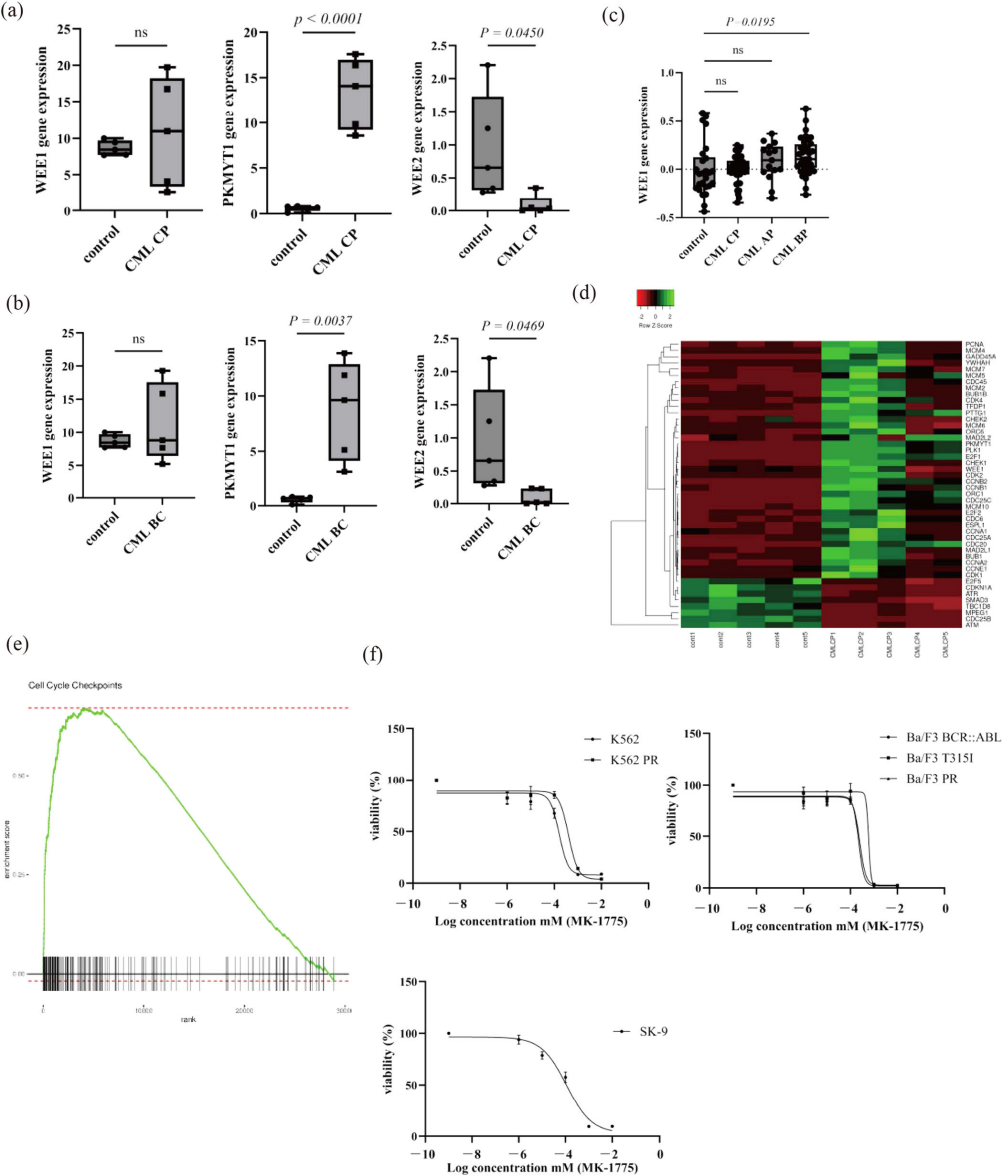
WEE1‐related gene expression in patients with CML and MK‐1775 activity in Ph‐positive cell lines. (a, b) Gene expression levels of *WEE1*, *PKMYT1*, and *WEE2* were confirmed through the GEO data set GSE100026 by comparing five healthy patient samples (control) with five samples each from chronic phase (CP) and blast phase (BP) patients with CML. (c) Gene expression levels of *WEE1* were confirmed through the GEO data set GSE4170 by comparing healthy patient samples (control) with patients with CML in the CP, AP, and BP. (d, e) Analyses of cell cycle‐regulating gene clusters and gene set enrichment were performed using the open bioinformatics web tools RaNA‐Seq and Heatmapper. (f) Ph‐positive cell lines were grown for 72 h in RPMI 1640 medium supplemented with 10% FBS and different concentrations of MK‐1775. Cell viability was assessed using the Cell Counting Kit‐8 assay.

Solid tumors, including hepatocellular carcinoma and colon cancer, exhibit elevated expression of *WEE1* and *PKMYT1* [10]. We first assessed *WEE1* expression in patients using publicly available GEO data. Our analysis using the GEO database (GSE100026) revealed that *PKMYT1* expression was elevated in both CML CP and BP samples compared with that in normal cells [17]. *WEE1* expression remained constant in both CML and healthy cells, but was reduced in the CML CP and BP samples (Figure 1a,b). Analysis of the GSE4170 data set revealed that *WEE1* expression was upregulated in patients with CML BP, suggesting a potential role for *WEE1* in disease progression (Figure 1c). Hierarchical clustering analysis produced a heatmap revealing increased expression of *PKMYT1* in patients with CML compared with healthy individuals (Figure 1d). Analysis of the RNA sequencing data revealed changes in the expression of genes involved in cell cycle checkpoint regulation (Figure 1e). MK‐1775 is a small molecule that acts as a potent inhibitor of WEE1 kinase [11]. We examined the effects of MK‐1775 on the CML cell lines. The results showed that MK‐1775 markedly inhibited CML cell growth and was effective against Ph‐positive cell lines resistant to ponatinib or those carrying the T315I mutation (Figure 1f).

### 3.2 MK‐1775 Induced Ph‐Positive Cell Death

**Figure 2:**
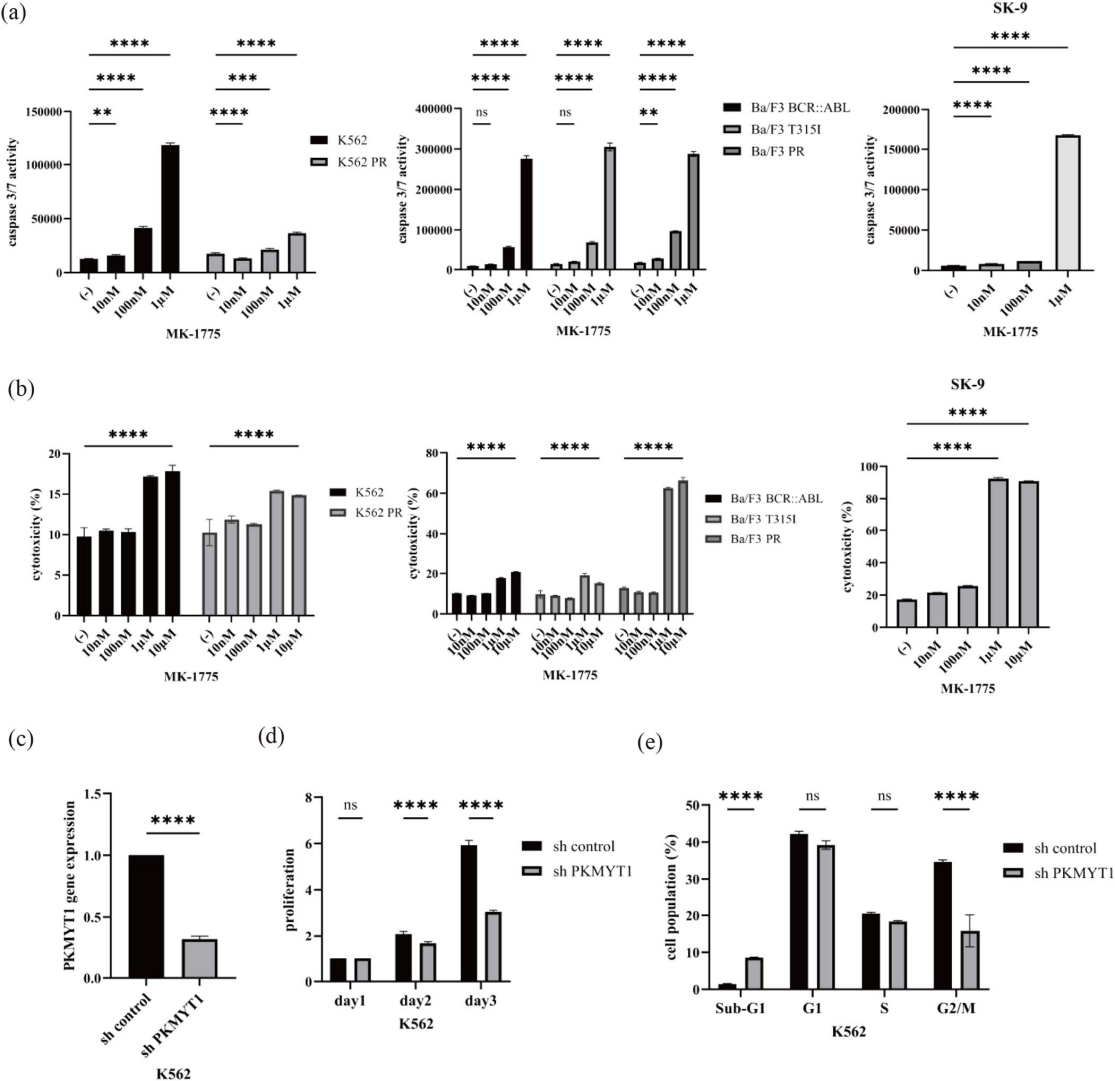
Effect of MK‐1775 on Ph‐positive cells. (a) Ph‐positive cells were incubated with specified concentrations of MK‐1775 for 48 h, after which caspase 3/7 activity was assessed. **p < 0.01, ***p < 0.001, and ****p < 0.0001 compared to control. (b) Ph‐positive cells were treated with MK‐1775 for 48 h. Subsequently, cytotoxicity was assessed using the Cytotoxicity LDH Assay Kit. ****p < 0.0001 versus control. (c) Gene expression of *PKMYT1* was evaluated using real‐time PCR analysis. Significance is expressed as ****p < 0.0001 versus the control shRNA transfectant cells. (d) Cellular proliferation of shRNA‐transfected K562 cells was evaluated using Trypan blue staining or Cell Counting Kit‐8. Significance is expressed as ****p < 0.0001 versus the control shRNA transfectant cells. (e) K562 cells transfected with shRNA were cultured for 24 h. The cell cycle phase distribution was analyzed using the BD Cycletest Plus DNA Reagent Kit. Representative histograms for each condition are shown. Statistical significance is indicated as ***p < 0.001, ****p < 0.0001 versus control. ns, not significant.

In mammalian cells, apoptosis, the first known form of regulated cell death, is mediated by caspase 3/7 [22]. Cytotoxicity denotes the potential of a substance to induce cellular harm or death. We evaluated caspase 3/7 activity and cytotoxicity in MK‐1775 cells. Our results suggested that MK‐1775 triggers apoptosis in CML cells, as demonstrated by the elevated caspase 3/7 activity. The application of MK‐1775 led to a notable increase in both caspase 3/7 activity and cytotoxicity, suggesting an increase in apoptotic cell death among treated cells (Figure 2a,b). The therapeutic potential of MK‐1775 in CML was further supported by the observed increase in caspase 3/7 activity and induction of cell death upon treatment. Notably, MK‐1775 demonstrated efficacy against T315I‐mutant and ponatinib‐resistant CML cell lines, highlighting its promise as a novel therapeutic option for drug‐resistant CML. To investigate the functional role of *PKMYT1*, K562 cells were transfected with shRNA targeting *PKMYT1*. A marked reduction in *PKMYT1* expression was observed in shRNA‐transfected cells compared with control shRNA‐transfected cells (Figure 2c). This knockdown was associated with a significant decrease in cell proliferation (Figure 2d). Cell cycle analysis further revealed an increase in the sub‐G1 population and a concomitant decrease in the G2/M phase in *PKMYT1*‐depleted cells (Figure 2e), suggesting enhanced apoptosis and impaired cell cycle progression.

### 3.3 VK2 Activity in CML Cells

**Figure 3:**
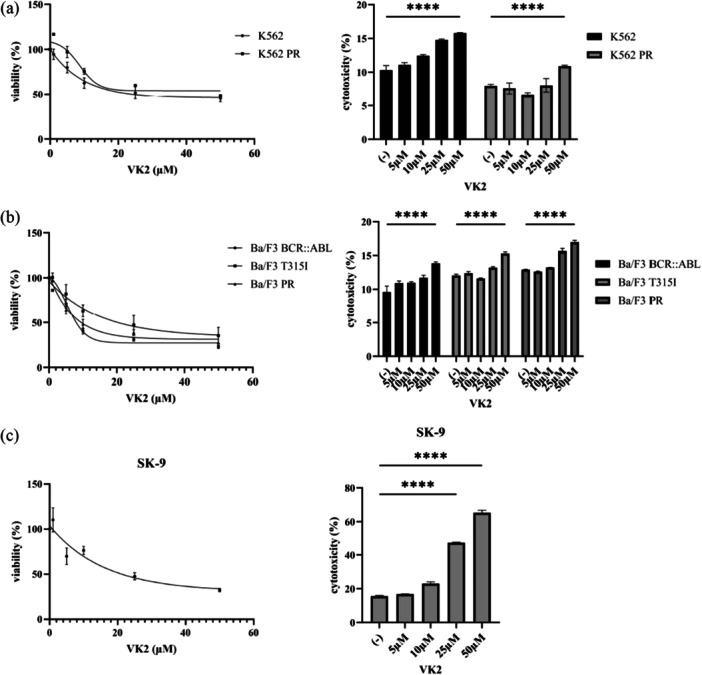
Effect of VK2 on Ph‐positive cells. (a–c) Ph‐positive cells were treated with VK2 for 48 or 72 h. Cell growth was measured using the CellTiter‐Glo Luminescent Cell Viability Assay Kit or the Cell Counting Kit‐8, and cytotoxicity was evaluated accordingly. ****p < 0.0001 versus control. ns, not significant.

VK2, a versatile nutrient essential for human health, has recently become the focus of numerous studies, which have shown that it exhibits anticancer activity in various types of cancer cells [13]. Therefore, we next examined the possible anticancer effects of VK2 in CML cells resistant to ABL inhibitors. Our study showed that VK2 suppressed the growth of CML cell lines in proportion to the administered dose (Figure 3a–c). Higher concentrations of VK2 significantly decreased cell viability, demonstrating the ability of VK2 to inhibit CML cell growth. Additionally, the cytotoxic effects of VK2 became more pronounced as the treatment levels increased, indicating that it triggered cell death through toxic mechanisms. These results highlight VK2's potential as a therapeutic target for ABL TKI‐resistant cells.

### 3.4 MK‐1775 and VK2 Inhibited Colony Formation of Ph‐Positive Cells

**Figure 4:**
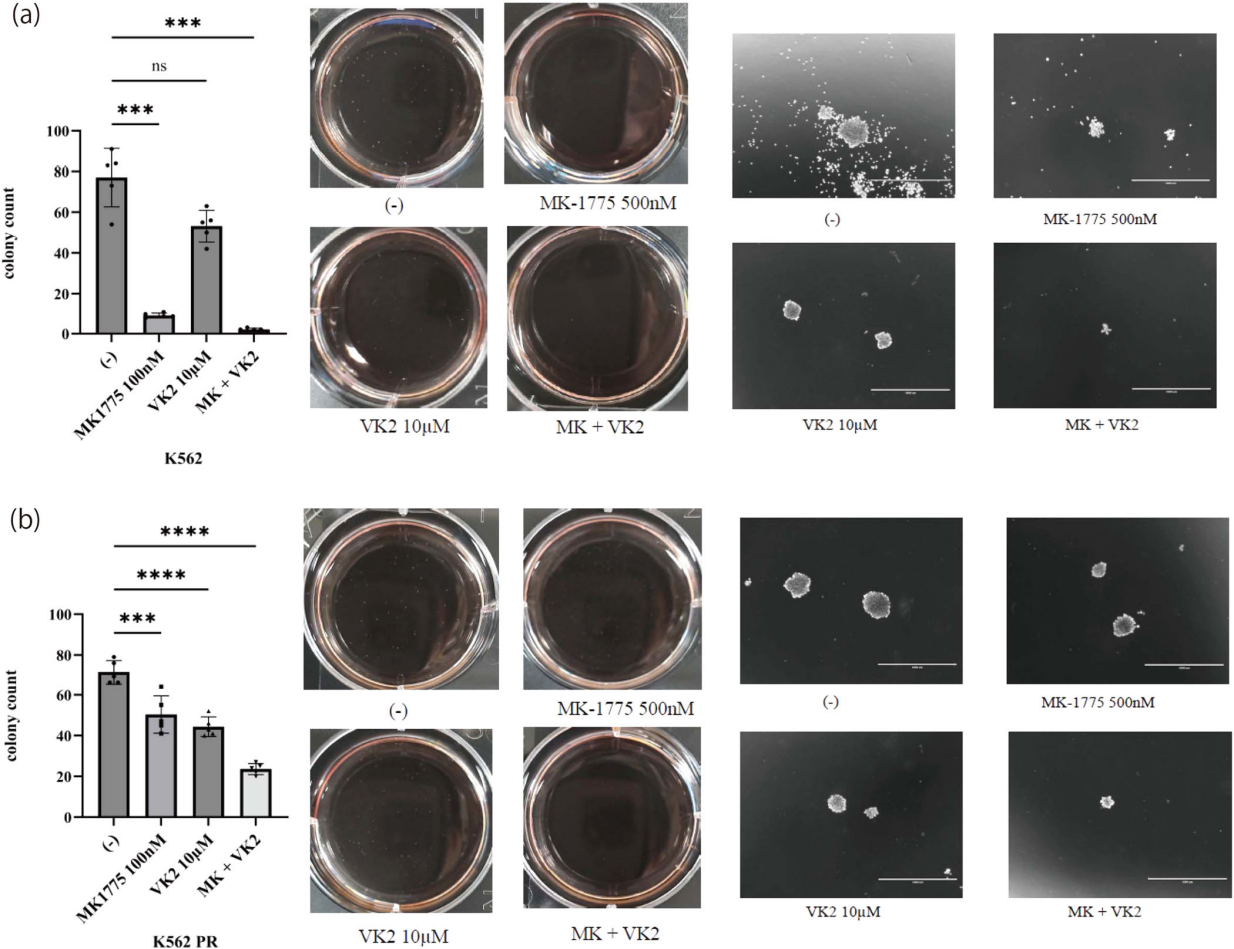
Colony formation assays in CML cell lines. (a, b) CML cell lines were treated with 500 nM MK‐1775 and/or 10 μM VK2 for 7–9 days. The colonies were captured using a digital camera and quantified using an EVOS FL Digital Inverted Fluorescence Microscope. The quantification graph illustrates colony formation using representative images from three separate experiments. The scale bar was set at 1000 μm, and the results are representative of three independent experiments. ***p < 0.001, ****p < 0.0001 versus control. ns, not significant. (‐) represents the untreated control.

Colony‐forming assays were performed using various CML cell lines to investigate the specific effects of MK‐1775 and VK2 on the viability and growth of CML cells. We aimed to assess the long‐term impact of combined treatment on the colony‐forming ability of CML cells and to measure their survival and proliferation potential. The results revealed that the combination of MK‐1775 and VK2 significantly reduced colony formation in both K562 and ponatinib‐resistant K562 PR cells (Figure 4a,b). These findings revealed that the colony size was reduced by treatment with MK‐1775 and VK2. These observations suggest that combination therapy hinders the ability of CML cells to survive and multiply over an extended period, even in cell lines that develop drug resistance.

### 3.5 MK‐1775 and VK2 Inhibited the Proliferation of Ph‐Positive Cells

**Figure 5:**
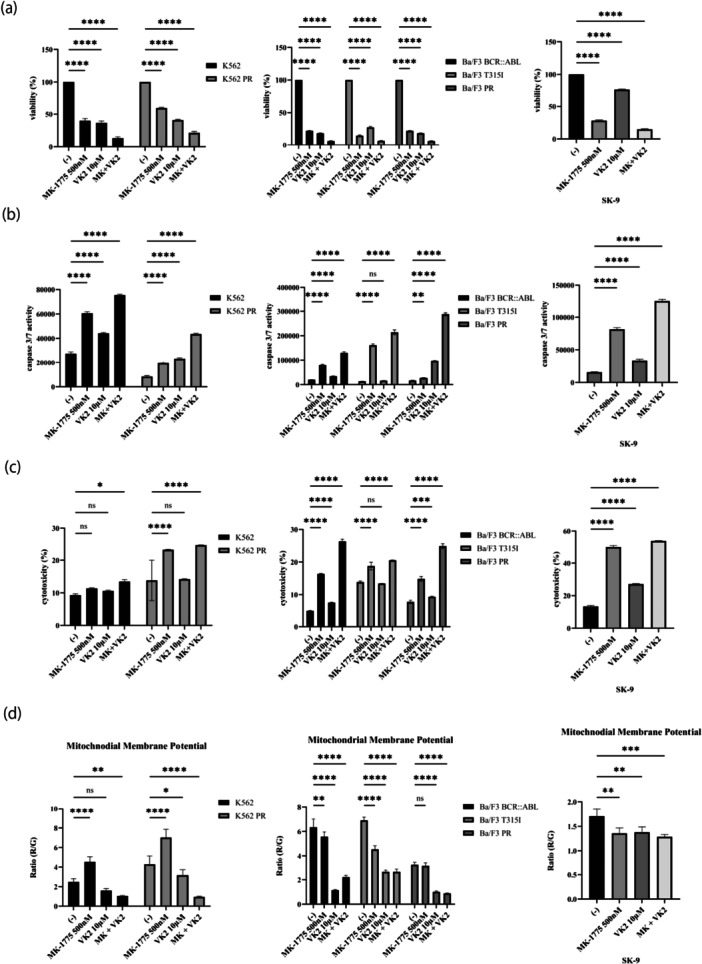
Co‐treatment of Ph‐positive cell lines with MK‐1775 and VK2. (a) Ph‐positive cells were treated with MK‐1775 and/or VK2 for 72 h, and the cell viability was assessed. ****p < 0.0001 versus control. (b) Ph‐positive cells were treated with MK‐1775 and/or VK2 for 48 h, and caspase 3/7 activity was analyzed. **p < 0.01 and ****p < 0.0001 versus control, ns, not significant. (c) Ph‐positive cells were treated with MK‐1775 and/or VK2 for 48 h. Cytotoxicity was evaluated using the Cytotoxicity LDH Assay Kit. *p < 0.05, ***p < 0.001, and ****p < 0.0001 versus control. ns, not significant. (d) Ph‐positive cells were treated with MK‐1775 and/or VK2 for 48 h. MMP was examined using a mitochondrial staining kit. *p < 0.05, **p < 0.01, ***p < 0.001, and ****p < 0.0001 versus control. ns, not significant.

We examined the combined effects of MK‐1775 and VK2 in Ph‐positive cell lines. The results demonstrated that the simultaneous administration of MK‐1775 and VK2 resulted in greater inhibition of Ph‐positive cell growth than the use of either compound independently (Figure 5a). Figure S1 shows that the drug combination exhibited enhanced efficacy. To further validate this observation, drug combination studies were conducted using the Chou–Talalay method to quantitatively assess drug interactions [21]. The calculated CI values were consistently less than 1, indicating a synergistic effect between the compounds tested (data not shown). We have provided Figure S1 to ensure transparency and support our findings. In Ph‐positive cell lines, combined treatment markedly elevated caspase 3/7 activity and amplified cytotoxic effects (Figure 5b,c). MMP, an essential factor in energy storage during oxidative phosphorylation, is generated by proton pumps, particularly Complexes I, III, and IV [23]. Next, we examined the MMP activities of MK‐1775 and VK2. Our results revealed a significant reduction in MMP after the administration of the combination treatment, implying that MK‐1775 and VK2 impair mitochondrial activity, potentially triggering cellular stress or programmed cell death (Figure 5d). Our observations indicate possible interference with the cell's energy production processes and the initiation of cellular demise.

## 4 Discussion

We examined the potential medicinal benefits of MK‐1775 and VK2, both independently and in combination, in Ph‐positive cell lines. The findings revealed promising results regarding their ability to suppress cellular growth, trigger programmed cell death, and boost toxicity toward cancer cells, particularly in those resistant to ABL TKIs.

This study is the first to investigate increased expression of WEE1 in multiple cancer types, including CML. As an essential regulator of the G2/M checkpoint, WEE1 prevents cells from prematurely entering mitosis and causes DNA damage [8]. The analysis of publicly available gene expression data (GEO data set GSE100026) revealed elevated levels of *PKMYT1*, a gene linked to WEE1, in patients with CML compared with healthy controls. Our analysis of the GSE4170 data set further revealed that *WEE1* expression was significantly upregulated in patients with CML BP, suggesting a potential role for WEE1 in disease progression. Our findings indicate that MK‐1775 exerts strong antileukemic effects in CML, including T315I‐mutant and ponatinib‐resistant cells, through the induction of apoptosis and cell cycle disruption. *PKMYT1* knockdown reduced proliferation and promoted cell death, suggesting its role in the mechanism of action of MK‐1775. These results highlight the potential of MK‐1775 as a treatment for drug‐resistant CML and support further investigation of VK2 as a complementary therapeutic approach. We demonstrated that MK‐1775 successfully inhibited the growth of CML cells, including those with the T315I mutation and ponatinib‐resistant cell lines. This finding is particularly important because the T315I mutation is notorious for its high resistance to conventional TKIs [24], and ponatinib‐resistant CML presents a significant therapeutic challenge. The effectiveness of MK‐1775 in inhibiting the growth of resistant cells shows its promise as a new therapeutic approach for drug‐resistant CML.

VK2 treatment resulted in a dose‐dependent suppression of CML cell proliferation, with higher concentrations of VK2 leading to a marked decrease in cell viability and an increase in cytotoxicity. These findings suggest that VK2 triggers cell death through apoptotic mechanisms, although the exact mechanism remains unclear. The potential of VK2 as an anticancer agent is particularly significant in CML, where resistance to standard therapies, such as TKIs, poses a considerable challenge. The findings of this study suggest that VK2 may serve as an effective therapeutic option, either alone or in combination with other agents such as MK‐1775, a selective WEE1 kinase inhibitor that is critical for controlling the G2/M cell cycle checkpoint [11]. When WEE1 is inhibited, the checkpoint is removed, causing the cells with DNA damage to undergo mitosis. This process results in cell death, particularly in tumor cells deficient in p53 [11]. Several clinical studies on MK‐1775 have been published. MK‐1775 administered daily at a dose of 200 mg demonstrated good tolerability in Japanese patients with advanced solid tumors. The drug had a manageable safety profile, limited anticancer activity, and pharmacokinetics that were slightly more than dose‐proportional [25]. A review of data from eight Phase I/II studies showed that body weight, renal impairment, and race significantly impacted MK‐1775 exposure, as revealed by population pharmacokinetic modeling. These factors are recognized as major determinants of drug behavior within the body [26]. The AcSé‐ESMART Arm C trial showed that combining MK‐1775 with carboplatin resulted in significant blood‐related adverse effects. However, the study indicated potential efficacy in childhood cancers with specific genetic alterations, highlighting the necessity for further research to explore less harmful treatment strategies [27].

The combination of MK‐1775 and VK2 has demonstrated significant effectiveness in impairing mitochondrial function, inducing programmed cell death, and enhancing toxicity in CML cells, including in drug‐resistant variants. This synergistic approach could offer a more robust treatment option, particularly for patients with CML who no longer respond to TKI therapy. Targeting mitochondrial processes, along with the ability of MK‐1775 to inhibit the G2/M checkpoint, presents a promising strategy to overcome drug resistance and promote cell death in CML cells.

Although the combination of MK‐1775 and VK2 demonstrated enhanced antileukemic effects in vitro, the potential for toxicity—particularly hematologic adverse events reported in previous clinical trials involving MK‐1775—must be carefully considered. Although no apparent cytotoxicity was observed under the experimental conditions of this study, the safety profile of the MK‐1775/VK2 combination remains to be fully elucidated. Moreover, as this study was limited to in vitro analyses, in vivo validation using patient‐derived xenograft models or syngeneic mouse models will be essential to assess the efficacy and safety of this combination in a physiological context. Future studies are planned to establish appropriate in vivo models to confirm the translational potential of this therapeutic strategy.

There are some limitations of this research. The present study lacked in vivo validation because all experiments were conducted using CML cell lines. Additionally, the precise molecular mechanisms underlying the synergistic effects of MK‐1775 and VK2 remain unclear. Preclinical and clinical studies are needed to assess the safety and efficacy of this combination for CML.

## 5 Conclusions

In this study, we investigated the potential therapeutic benefits of MK‐1775 and VK2 in treating CML. MK‐1775 effectively decreased cell proliferation and promoted apoptosis in CML cells, including those resistant to the drugs. VK2 exhibited anticancer effects that varied with dosage. When used together, MK‐1775 and VK2 produced synergistic outcomes, leading to greater inhibition of CML cell growth, enhanced apoptosis, and decreased MMP. These results indicated that MK‐1775 and VK2, used individually or in combination, could offer promising new treatment options for CML, especially in cases of drug resistance.

## Author Contributions

**Seiichi Okabe:** conceptualization (equal), investigation (equal), writing – original draft (lead). **Yuya Arai:** conceptualization (equal), investigation (equal). **Akihiko Gotoh:** project administration (lead), writing – review and editing (equal). **Daigo Akahane:** project administration (supporting), supervision (equal).

## Ethics Statement

The authors have nothing to report.

## Consent

The authors have nothing to report.

## Conflicts of Interest

A.G. received research funding from Eisai Co. Ltd., Ono Pharmaceutical Co. Ltd., Taiho Pharmaceutical Co. Ltd., Takeda Pharmaceutical Co. Ltd., Nippon Shinyaku Co. Ltd., and Chugai Pharmaceutical Co. Ltd.; MSD K.K.; Otsuka Pharmaceutical Co. Ltd., Sumitomo Pharma Co. Ltd., Nippon Shinyaku Co. Ltd., Bayer Yakuhin Ltd., Daiichi Sankyo Co. Ltd., and Nihon Pharmaceutical Co. Ltd.; A.G. received honoraria from Novartis Pharma K.K., Alexion Pharmaceuticals Inc., Eisai Co. Ltd., Ono Pharmaceutical Co. Ltd., Taiho Pharmaceutical Co. Ltd., Takeda Pharmaceutical Co. Ltd., Nippon Shinyaku Co. Ltd., Chugai Pharmaceutical Co. Ltd., Otsuka Pharmaceutical Co. Ltd., Sumitomo Pharma Co. Ltd., Daiichi Sankyo Co. Ltd., Nihon Pharmaceutical Co. Ltd., Kyowa Kirin Co. Ltd., Janssen Pharmaceutical K.K., Pfizer Japan Inc., and Sanofi K.K.; A.G. received consulting fees from PharmaEssentia Japan K.K., Chugai Pharmaceutical Co. Ltd., and Alexion Pharmaceuticals Inc. Additionally, A.G. participated on the data safety monitoring board or advisory board of PharmaEssentia Japan K.K., Chugai Pharmaceutical Co. Ltd., and Alexion Pharmaceuticals Inc. S.O., Y. A. and D.A. declare no conflicts of interest.

## Supporting information


**Figure 1:** Activity of MK‐1775 and VK2 in CML cell lines.

## Data Availability

The data sets generated and/or analyzed during this study are available from the corresponding author upon reasonable request.
