# Supplementary material for: Combination of WEE1 Inhibitor and Vitamin K2 Enhances Therapeutic Efficacy in Chronic Myeloid Leukemia
Source: Cancer Innov. 2025 Aug 28;4(5):e70024. doi: 10.1002/cai2.70024 (PMC12394061; doi:10.1002/cai2.70024)
Supplement: Supplementary file 1 — Figure 1: Activity of MK‐1775 and VK2 in CML cell lines. [file CAI2-4-e70024-s001.doc]

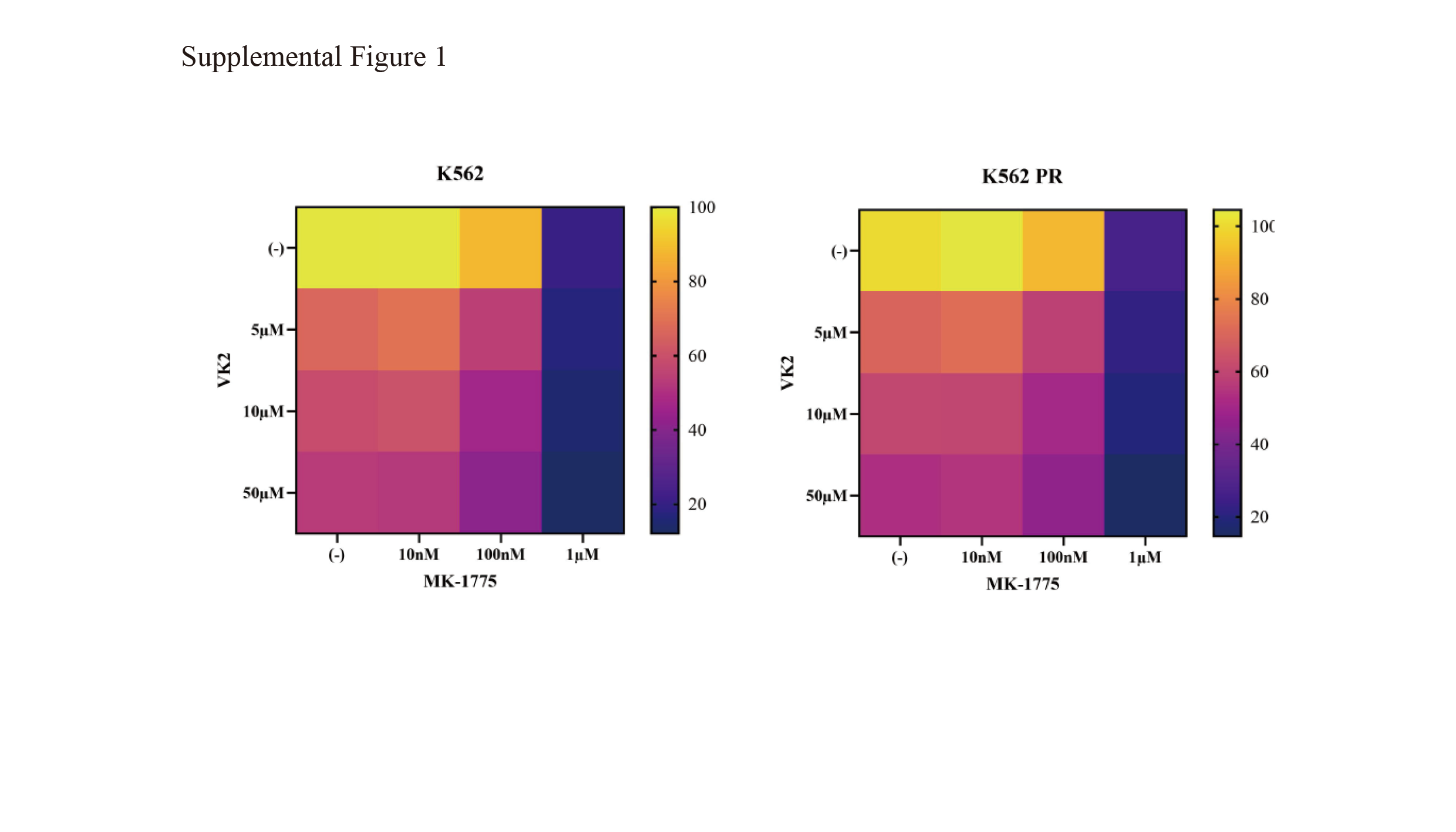
**Supplemental Figure 1.** Activity of MK-1775 and VK2 in CML cell lines.

K562 and K562 PR cells were treated with MK-1775 and/or VK2 for 72 h. The cell viability was evaluated.
